# Characteristics of Patients and Treatment Recommendations from a Multidisciplinary Spinal Tumor Program

**DOI:** 10.1089/pmr.2020.0070

**Published:** 2020-07-31

**Authors:** Mai Anh Huynh, Claudia Roldan, Paula Nunes, Andrea Kelly, Allison Taylor, Cara Richards, M. Mohsin Fareed, Daniel Gorman, Michael Groff, Marco Ferrone, Yi Lu, John H. Chi, Alexander Spektor, Tracy Balboni

**Affiliations:** ^1^ Department of Radiation Oncology, Dana-Farber/Brigham and Women's Cancer Center, Boston, Massachusetts, USA.; ^2^ Department of Adult Palliative Care, Dana-Farber Cancer Institute, Boston, Massachusetts, USA.; ^3^ Department of Orthopedic Surgery, and Dana-Farber/Brigham and Women's Cancer Center, Boston, Massachusetts, USA.; ^4^ Department of Neurosurgery, Dana-Farber/Brigham and Women's Cancer Center, Boston, Massachusetts, USA.

**Keywords:** palliative care, radiosurgery, spinal neoplasms

## Abstract

***Objective:*** We describe characteristics of patient and treatment recommendations from a spinal tumor board at one institution, including representation from palliative care.

***Background:*** The impact of prospective multidisciplinary input for patients with spinal tumors is poorly understood despite their increasing complexity.

***Methods:*** We retrospectively reviewed 622 cases sequentially discussed at a weekly spinal tumor board, and abstracted patient and treatment information from the medical record and meeting minutes.

***Results:*** From April 2017 to February 2019, 622 cases representing 438 unique patients were discussed. The median age was 62 years (range 21–92). Most patients had spinal tumors originating from metastases (91.78%), including breast (14.3%), nonsmall cell lung cancer (13.4%), prostate (10.9%), and renal cell cancer (8.8%), and the remainder had primary central nervous system (4.3%) or benign tumors (3.9%). Sixty-five percent of patients were alive at last follow-up. Conventional external beam radiotherapy was the most common treatment recommendation (33.8%) followed by surgery (26.2%), stereotactic body radiation therapy (17.8%), imaging follow-up (16.6%), and vertebroplasty (15.9%). Palliative care was the primary treatment recommended for 4.5%, and no therapy recommended for 4.0%. Treatment recommendation involved two modalities for 29% of cases, and three in 1.3% of cases. In four cases, biopsy to confirm pathology changed management due to unexpected findings of osteomyelitis, hematopoiesis, or new diagnosis of plasmacytoma.

***Conclusions:*** Multidisciplinary input is integral to the optimal care of spinal tumor patients. The high risk of death highlights the need to prioritize modalities that optimize quality of life in the context of a patient's individual prognosis.

## Introduction

Spinal metastases arise frequently for ∼5%–30% of patients with a primary cancer, with the most common originating from the breast, lung, kidney, and prostate.^1,2^ Patients with spinal tumors often experience pain, neurologic symptoms, biomechanical instability, and bone fractures, which can significantly decrease quality of life and life expectancy.^3,4^ Metastatic spinal disease is considered a terminal stage of primary cancer and palliative treatment centers around symptom control and reducing debilitating spinal complications such as cord compression. Given the broad range of clinical presentations and complexity of symptoms, the optimal management of spinal tumors involves consideration of neurologic, oncologic, mechanical, and systemic features.^5^

Treatment decisions for patients with spinal tumors have become more complex in an era of new and improved systemic and local treatments, as patients survive long enough to experience morbidity from tumor progression or risk complications from prior therapies. The treatment of spinal tumors has evolved from conventional external beam radiation therapy (cEBRT) as the primary modality of treatment, with or without upfront surgery for symptomatic cord compression,^2,6^ to include an array of treatment options that require a more nuanced understanding of the competing risks of local and distant progression and multimodal assessments that integrate medical oncology, radiation oncology, surgery, radiology, and palliative care expertise.^5^

Patients with a more favorable prognosis may benefit from stereotactic body radiation therapy (SBRT), which allows for delivery of high doses of radiation to the target while minimizing radiation exposure to nearby critical nerve structures, and has been associated with higher rates of local control (LC).^7,8^ Depending on the proximity of the epidural tumor to spinal cord or in the setting of reirradiation, separation surgery with the intent of creating greater separation between the tumor and spinal cord to allow for SBRT may be the optimal treatment option to achieve more durable LC.^9^ For patients with pathologic compression fractures, minimally invasive vertebroplasty/kyphoplasty may help to alleviate symptoms of mechanical pain or augment spinal stability.^10^ Vertebroplasty/kyphoplasty before or after SBRT may also reduce risk of symptomatic fracture after SBRT for patients at high risk of vertebral compression fracture.^11^ Cryoablation may be an option for patients with spinal tumors that have not responded to radiation treatment. Local nerve block or steroid injection may also be options to address symptoms, when surgery or radiation treatment is not a viable option. Spinal tumor boards, therefore, allow an opportunity for efficient clinical and radiographic review and a forum for collaborative decision making, while also refining the intent of local therapy in the context of a patient's competing risks of systemic progression and goals of care.

Established in 2011, our institutional spinal tumor program is one of the first of its kind in the country and uniquely includes representation from neuroradiology, orthopedic and neurosurgery, radiation oncology, and adult palliative care to build consensus regarding optimal treatment for patients with spinal tumors at various time points in their continuum of care. Multidisciplinary teams are considered best practice in cancer care and there is ample data showing improved clinical outcomes and greater cooperation among medical teams.^12,13^ There are only a few spinal tumor board programs at various academic medical centers throughout the nation in which clinicians who are experts in different specialties meet to tailor treatment, incorporating spine oncology expertise. The disciplines represented in the tumor boards vary by institution, but medical oncologists, surgeons, and radiation oncologists are usually represented and less commonly include representation from adult palliative care.

Despite the increasing need for expert multidisciplinary expertise in the care of spinal tumor patients, little is known about the treatment recommendations, patient characteristics, and clinical outcomes of multidisciplinary spinal tumor board programs and the potential value they may add to patient care. Therefore, the purpose of this study was to characterize the patient and treatment recommendations and clinical outcomes of patients discussed at a weekly multidisciplinary spinal tumor board to better understand the impact of multidisciplinary input in the care of this complex patient population and inform future clinical decision making.

## Materials and Methods

A multidisciplinary spinal tumor program including radiation oncology, neuroradiology, orthopedic surgery, neurosurgery, and palliative care providers convened weekly to review complex spine cases and formulate a consensus recommendation at an academic institution and tertiary referral center for oncology and spinal tumor patients.

Patients reviewed included inpatients and outpatients referred from colleagues in medical oncology, radiation oncology, surgical oncology, palliative care, and from among patients seen by tumor board members, including patients without a known oncologic diagnosis. The clinical history, symptoms and examination, prior radiation and surgical history, and imaging to review and clinical question were submitted by either core attendees or referring providers.

Treatment options were discussed in a multidisciplinary forum until consensus was achieved regarding optimal treatment options, which incorporated information regarding prognosis, performance status, or goals of care when able based on medical record or further discussion with primary team. These recommendations were communicated to referring providers to facilitate appropriate multidisciplinary follow-up, imaging, or work-up. Patients were reviewed at spinal tumor board at consecutive conferences until a treatment plan was satisfactorily in place, for example, in cases of diagnostic uncertainty wherein further diagnostic imaging or pathology review was needed and before and after surgical decompression to facilitate multidisciplinary transitions in care.

After obtaining institutional review board approval, we performed a retrospective analysis of 438 patients who were discussed at our spinal tumor program from April 2017 to February 2019. Patient clinical information, tumor characteristics, and consensus recommendations were abstracted from electronic medical records and analyzed.

## Results

### Case conference characteristics

There were 622 total presentations representing 438 unique patients from April 2017 to February 2019 at a single academic medical center (Table 1). The median number of presentations per patient was 1.42 (range: 1–7), with 388 patients discussed 1 time, 88 patients on 2 occasions, 44 patients on 3 to 4 separate occasions, and 5 patients discussed on 6 to 7 separate occasions. Conventional radiotherapy (RT) was the most common treatment recommendation (33.8%) followed by surgery (26.2%), SBRT (17.8%), imaging follow-up (16.6%), and vertebroplasty/kyphoplasty (15.9%). Multimodal treatments that required at least two modalities comprised 29% while those requiring at least three modalities comprised 1.3% (Table 2).

**Table 1.** Summary of Case Conference Characteristics from April 2017 to February 2019

Case conference characteristics	n
Total patient presentations	622
Unique patients presented	438
Average per patient	1.42
Range per patient	1,7

**Table 2.** Summary of Consensus Recommendations by Discipline and Frequency of Multimodality Treatment

Treatment recommendation	Total (n = 622)	Percent
Conventional radiation	210	33.8
SBRT	111	17.8
Surgery	163	26.2
Biopsy	58	9.3
Vertebroplasty/kyphoplasty	99	15.9
Nerve injection	5	0.8
Palliative care	28	4.5
Imaging follow-up	103	16.6
Medical oncology referral	33	5.3
Cryoablation	23	3.7
None	25	4.0
Multimodal treatment		
At least two modalities	181	29
At least three modalities	8	1.3

SBRT, stereotactic body radiation.

### Patient and tumor characteristics

The median age of patients was 62 years (range: 21–92), with an almost equal distribution of men (49.1%) and women (50.9%). At the time of data analysis, 302 (64.5%) of patients were alive. The majority of patients discussed had metastatic spinal tumors originating from a wide range of primary sites, 402 (91.78%), whereas 19 (4.34%) presented with a primary central nervous system tumor and 17 (3.88%) had a benign tumor. Among the patients presenting with metastases, the most common primary cancers were 67 (14.3%) breast cancer, 65 (13.9%) nonsmall lung cancer, 51 (10.9%) prostate cancer, 40 (8.5%) renal cell cancer (RCC), 30 (6.4%) hematologic cancer (i.e., lymphoma, multiple myeloma, and plasmacytoma), and 28 (6%) sarcoma. Other primary histologies represented included 16 (3.4%) colorectal cancer, 15 (3.2%) melanoma, 12 (2.6%) head and neck cancer, and 9 (1.9%) bladder cancer among a wide but smaller variety of primary cancers (Table 3). In four cases, biopsy intended for pathologic confirmation of presumed metastatic involvement changed management of disease by revealing an alternative etiology for radiographic changes, including for one patient with osteomyelitis, two patients with hematopoiesis, and one patient with recurrent ovarian cancer diagnosed with new solitary plasmacytoma.

**Table 3.** Tumor Characteristics among Patients Presented at Spinal Tumor Board

Tumor characteristics	n	Percent
Breast	67	14.3
NSCLC	65	13.9
Prostate	51	10.9
Renal cell cancer	40	8.5
Heme	30	6.4
Sarcoma	28	6
Bladder	9	1.9
Germ cell tumor	1	0.2
Seminoma	1	0.2
Carcinoid	1	0.2
Mesothelioma	1	0.2
SCLC	7	1.5
Thymoma	1	0.2
Melanoma	15	3.2
Head and neck	12	2.6
Thyroid	7	1.5
Cervical	1	0.2
Endometrial	4	0.9
Ovarian	2	0.4
Colorectal	16	3.4
Esophageal	7	1.5
Gastric	1	0.2
GIST	1	0.2
HCC	2	0.4
Cholangiocarcinoma	5	1.1
Pancreatic	4	0.9
Carcinoma of unknown primary	1	0.2
Unknown	3	0.6
Primary CNS		
Chordoma	3	0.6
Ependymoma	3	0.6
Glioblastoma	1	0.2
Meningioma	2	0.4
MPNST	4	0.9
Paraganglioma	1	0.2
Schwannoma	4	0.9
Melanocytoma	1	0.2
Benign		
Hemangioblastoma	1	0.2
Hemangioendothelioma	1	0.2
Hemangioma	1	0.2
Lipoma	2	0.4
Neuroendocrine	8	1.7
Pheochromocytoma	3	0.6

GIST, gastrointestinal stromal tumor; HCC, hepatocellular carcinoma; heme, hematologic cancer (i.e., lymphoma, multiple myeloma, and plasmacytoma); MPNST, malignant peripheral nerve sheath tumor; NSCLC, nonsmall lung cancer; SCLC, small cell lung cancer.

Between April 2017 and February 2019, there were 75 unique patients treated at our institution with radiation for true malignant epidural spinal cord compression (high-grade ESCC), most often in the postoperative setting (51%) unless the patient had a radiosensitive histology or because surgery was not within their goals of care. Of those, 37 were discussed at spine tumor program (49%). In this same time period, there were 86 unique patients treated at our institution with radiation with nerve root compression or low-grade epidural cord compression. Of those, 25 were also discussed at spine tumor program (29%).

## Discussion

To our knowledge, this is the first report of patient and tumor characteristics and treatment recommendations from a multidisciplinary spinal tumor board program. The range in patient age, tumor histologies, and the high proportion of treatment recommendations involving multimodal care presented at the board highlights both the prevalence of spinal disease and the complexity underpinning each individual case.^14^ Through multidisciplinary discussion, treatment recommendations were able to be efficiently and systematically optimized based on patient and tumor-specific factors.

Conventional external beam radiation treatment as monotherapy was favored for patients with uncomplicated spine metastases, without impending or existing pathologic fracture or existing spinal cord or cauda equina compression. Tumor histology was critical in determining whether cEBRT was appropriate. Patients with known or suspected myeloma were not recommended for surgery given known radiosensitive histology and anticipated response to chemotherapy.^15^ Surgery could also be avoided for patients anticipated to have a favorable response to radiation, including newly diagnosed metastatic prostate or breast cancer.^16,17^ For patients presenting with radioresistant tumors from primaries such as RCC, conventionally fractionated RT alone has been shown to lead to suboptimal LC.^18,19^ RT response for patients with RCC spine metastases has also been associated with a shorter duration of response and poorer survival compared with those with more responsive tumors such as breast cancer.^20^ Given the advances of targeted therapies and improved overall survival rates for this patient population, addressing bone metastases with more modalities that can improve LC is necessary,^21,22^ and this patient population was frequently offered separation surgery followed by SBRT.

Spinal instability was a frequent consideration and the Spinal Instability Neoplastic Score (SINS)^23^ and albumin were among clinical features considered to determine whether surgical evaluation or intervention with vertebroplasty/kyphoplasty was indicated, frequently as an adjunct to radiation therapy. The extent of surgical approach could also be tailored to the oncologic situation, with more minimally invasive approaches when goal was separation to allow more room for radiation dose compared with *en bloc* resection in settings, such as sarcoma, where curative intent justified a more radical approach. Furthermore, performance status or preoperative albumin was also common features to factor into the risk of postoperative complication.

In cases wherein prognosis or goals of care could not be easily ascertained, further input from the patient's primary oncologist was sought. Our multidisciplinary forum also provided the opportunity to, at times, redirect treatment recommendations based on histology, individual patient status, and prognostication factors. For example, patients with myeloma were not recommended for surgery given known radiosensitive histology and anticipated response to chemotherapy,^15^ whereas patients with suspected sarcoma were specifically guided to obtain biopsy, which would have significant implications on the surgical approach.^24^ Surgery could also be avoided for patients anticipated to have a favorable response to radiation, including newly diagnosed metastatic prostate or breast cancer, or to have dramatic response to chemotherapy, such as small cell lung cancer or myeloma.^15–17,25^ Conversely, more aggressive local therapy, including SBRT or separation surgery to allow for SBRT was most frequently recommended for patients with oligometastatic disease or to enable reirradiation.^26–28^ In addition, the high frequency of patients with multiple presentations at spine tumor program demonstrates the additional continuity of care such a forum could offer as patients progressed through various local treatments.

High-grade ESCC is an oncologic emergency. The known association of timely intervention with functional recovery for these patients required more urgent evaluation than the weekly spinal tumor board would allow.^29,30^ Approximately 50% of patients treated with radiation for high-grade ESCC between April 2017 and February 2019 were discussed at spine tumor program, likely reflecting the use of spine tumor board review for more complex cases where further discussion was indicated or to facilitate transitions in care. Approximately 29% of patients treated with radiation at our institution for palliation of symptomatic nerve root or lower grade epidural compression were discussed at the spinal tumor program. The opportunity for multidisciplinary review in this forum should not be a reason to delay initiation of care for patients with high-grade ESCC. However, it is possible that the relationships formed through the spinal tumor program lowered barriers for direct multidisciplinary discussion or consultation with spinal tumor experts before initiation of urgent treatment, although this cannot be quantified.

Our spinal tumor board integrates the NOMS (neurologic, oncologic, mechanical, and systemic) decision-making framework, which has been developed to incorporate the different considerations that affect the management of spinal tumors. Multidisciplinary spinal tumor boards provide an opportunity to clarify goals of care, guide completion of appropriate staging before planned interventions, coordinate transitions in care, and manage disease burden for a highly complex patient population among providers with wide-ranging expertise. It is also an opportunity to measure prognostication and risk of death to devise a customized treatment approach for each patient. Integrating an oncologic perspective for patients with spinal tumors is of vital importance to balance the competing risks of local therapies with systemic progression and their overall quality of life.

The purpose of our study is to offer descriptive data of a spinal tumor board program at an urban academic medical center. One limitation is that we are unable to effectively measure outcomes of the spinal tumor board and there is limited follow-up. Nonetheless, the spinal tumor board allows providers to efficiently assess a highly complex case in a multidisciplinary way while still providing timely care and treatment recommendations. This is especially critical for patients with neurologic complications.

A special feature of our program is that it incorporates specialists in palliative care to optimize symptom control for many patients with spinal metastases. Although the primary treatment recommendation was captured as “Palliative Care Referral” for only 25 patients (4%) when surgery, radiation, or other intervention was not an option (Table 2), this does not capture the immense value that palliative care presence added to clinical decision making at spinal tumor program, symptom management for patients, or to facilitating discussions of goals of care for patients throughout their continuum of illness. The core involvement of an adult palliative care provider enabled direct referral for a large proportion of spinal tumor patients, many of whom were discussed on multiple separate occasions at spinal tumor program (33%). The palliative care provider was frequently the central link between the patient and other members of the multidisciplinary oncology team. Furthermore, the expertise of adult palliative care facilitated discussion and consideration of alternative pain management options, including epidural steroid injections or intrathecal catheter placement for pain control. In addition, the attendance of our institution's dedicated supportive and palliative radiation oncology (SPRO) team also helped to optimize communication with inpatient and outpatient referring oncology providers and facilitated the timely care for many patients in need of urgent radiation for symptom control.

Although it remains unclear whether discussion at spinal tumor program improves outcomes for patients, this model of multidisciplinary cancer care provides a process by which patients with metastatic spinal disease can also benefit from discussions incorporating a broad range of expertise. Given the complexity, high risk of death, and the various considerations of patients with spinal tumors, optimal care of these patients requires a multidisciplinary approach to treatment. A spinal tumor board program that incorporates specialists from medical oncology, radiation oncology, neurosurgery, orthopedic surgery, interventional neuroradiology, and palliative care is optimal to develop an individualized treatment plan for each patient and likely contributes to value in care in ways that remain to be characterized.

## Abbreviations Used

cEBRT: conventional external beam radiation therapy
ESCC: epidural spinal cord compression
LC: local control
RCC: renal cell cancer
RT: radiotherapy
SBRT: stereotactic body radiation therapy

## References

[1] Lee BH, Kim TH, Chong HS, et al.: Prognostic factor analysis in patients with metastatic spine disease depending on surgery and conservative treatment: Review of 577 cases. Ann Surg Oncol 2013;20:40–46 22956070 10.1245/s10434-012-2644-4

[2] Yao A, Sarkiss CA, Ladner TR, Jenkins , ALIII: Contemporary spinal oncology treatment paradigms and outcomes for metastatic tumors to the spine: A systematic review of breast, prostate, renal, and lung metastases. J Clin Neurosci 2017;41:11–23 28462790 10.1016/j.jocn.2017.04.004

[3] Hage WD, Aboulafia AJ, Aboulafia DM: Incidence, location, and diagnostic evaluation of metastatic bone disease. Orthop Clin N Am 2000;31:515–528 10.1016/s0030-5898(05)70171-1 11043092

[4] Hernandez RK, Wade SW, Reich A, et al.: Incidence of bone metastases in patients with solid tumors: Analysis of oncology electronic medical records in the United States. BMC Cancer 2018;18:44 29306325 10.1186/s12885-017-3922-0 PMC5756362

[5] Laufer I, Rubin DG, Lis E, et al.: The NOMS framework: Approach to the treatment of spinal metastatic tumors. Oncologist 2013;18:744–751 23709750 10.1634/theoncologist.2012-0293 PMC4063402

[6] Dunning EC, Butler JS, Morris S: Complications in the management of metastatic spinal disease. World J Orthop 2012;3:114–121 22919567 10.5312/wjo.v3.i8.114 PMC3425630

[7] Bishop AJ, Tao R, Rebueno NC, et al.: Outcomes for spine stereotactic body radiation therapy and an analysis of predictors of local recurrence. Int J Radiat Oncol Biol Phys 2015;92:1016–1026 26025777 10.1016/j.ijrobp.2015.03.037

[8] Mehta N, Zavitsanos PJ, Moldovan K, et al.: Local failure and vertebral body fracture risk using multifraction stereotactic body radiation therapy for spine metastases. Adv Radiat Oncol 2018;3:245–251 30202794 10.1016/j.adro.2018.04.002 PMC6128022

[9] Laufer I, Iorgulescu JB, Chapman T, et al.: Local disease control for spinal metastases following “separation surgery” and adjuvant hypofractionated or high-dose single-fraction stereotactic radiosurgery: Outcome analysis in 186 patients. J Neurosurg Spine 2013;18:207–214 23339593 10.3171/2012.11.SPINE12111 PMC5737630

[10] Ciftdemir M, Kaya M, Selcuk E, Yalniz E: Tumors of the spine. World J Orthop 2016;7:109–116 26925382 10.5312/wjo.v7.i2.109 PMC4757655

[11] Jawad MS, Fahim DK, Gerszten PC, et al.: Vertebral compression fractures after stereotactic body radiation therapy: A large, multi-institutional, multinational evaluation. J Neurosurg Spine 2016;24:928–936 26895526 10.3171/2015.10.SPINE141261

[12] Prades J, Remue E, van Hoof E, Borras JM: Is it worth reorganising cancer services on the basis of multidisciplinary teams (MDTs)? A systematic review of the objectives and organisation of MDTs and their impact on patient outcomes. Health Policy 2015;119:464–474 25271171 10.1016/j.healthpol.2014.09.006

[13] Rankin NM, Lai M, Miller D, et al.: Cancer multidisciplinary team meetings in practice: Results from a multi-institutional quantitative survey and implications for policy change. Asia Pac J Clin Oncol 2018;14:74–83 28949100 10.1111/ajco.12765

[14] Ecker RD, Endo T, Wetjen NM, Krauss WE: Diagnosis and treatment of vertebral column metastases. Mayo Clinic Proc 2005;80:1177–1186 10.4065/80.9.1177 16178498

[15] Bird JM, Owen RG, D'Sa S, et al.: Guidelines for the diagnosis and management of multiple myeloma 2011. Br J Haematol 2011;154:32–75 21569004 10.1111/j.1365-2141.2011.08573.x

[16] Hellerstedt BA, Pienta KJ: The current state of hormonal therapy for prostate cancer. CA Cancer J Clin 2002;52:154–179 12018929 10.3322/canjclin.52.3.154

[17] Ju DG, Yurter A, Gokaslan ZL, Sciubba DM: Diagnosis and surgical management of breast cancer metastatic to the spine. World J Clin Oncol 2014;5:263–271 25114843 10.5306/wjco.v5.i3.263 PMC4127599

[18] Onufrey V, Mohiuddin M: Radiation therapy in the treatment of me tastatic renal cell carcinoma. Int J Radiat Oncol Biol Phys 1985;11:2007–2009 2414257 10.1016/0360-3016(85)90285-8

[19] Wroński M, Maor MH, Davis BJ, et al.: External radiation of brain metastases from renal carcinoma: A retrospective study of 119 patients from the M. D. Anderson Cancer Center. Int J Radiat Oncol Biol Phys 1997;37:753–759 10.1016/s0360-3016(97)00006-0 9128947

[20] Maranzano E, Latini P: Effectiveness of radiation therapy without surgery in metastatic spinal cord compression: Final results from a prospective trial. Int J Radiat Oncol Biol Phys 1995;32:959–967 7607970 10.1016/0360-3016(95)00572-g

[21] Coppin C, Kollmannsberger C, Le L, et al.: Targeted therapy for advanced renal cell cancer (RCC): A Cochrane systematic review of published randomised trials. BJU Int 2011;108:1556–1563 21952069 10.1111/j.1464-410X.2011.10629.x

[22] Flanigan R, Campbell S, Clark J, Picken M: Metastatic renal cell carcinoma. Curr Treat Options Oncol 2003;4:385–390 12941198 10.1007/s11864-003-0039-2

[23] Fisher CG, DiPaola CP, Ryken TC, et al.: A novel classification system for spinal instability in neoplastic disease: An evidence-based approach and expert consensus from the Spine Oncology Study Group. Spine 2010;35:1221–1229 10.1097/BRS.0b013e3181e16ae2 20562730

[24] Rosenberg AE: Bone Sarcoma Pathology: Diagnostic Approach for Optimal Therapy. American Society of Clinical Oncology Educational Book American Society of Clinical Oncology Annual Meeting 2017;37:794–798 10.1200/EDBK_174697 28561653

[25] Tartarone A, Giordano P, Lerose R, et al.: Progress and challenges in the treatment of small cell lung cancer. Med Oncol 2017;34:110 28456992 10.1007/s12032-017-0966-6

[26] Ahmed KA, Barney BM, Davis BJ, et al.: Stereotactic body radiation therapy in the treatment of oligometastatic prostate cancer. Front Oncol 2012;2:215 23346551 10.3389/fonc.2012.00215 PMC3551203

[27] Myrehaug S, Sahgal A, Hayashi M, et al.: Reirradiation spine stereotactic body radiation therapy for spinal metastases: Systematic review. J Neurosurg Spine 2017;27:428–435 28708043 10.3171/2017.2.SPINE16976

[28] Palma DA, Olson R, Harrow S, et al.: Stereotactic ablative radiotherapy versus standard of care palliative treatment in patients with oligometastatic cancers (SABR-COMET): A randomised, phase 2, open-label trial. Lancet 2019;393:2051–2058 30982687 10.1016/S0140-6736(18)32487-5

[29] Bilsky MH, Laufer I, Fourney DR, et al.: Reliability analysis of the epidural spinal cord compression scale. J Neurosurg Spine 2010;13:324–328 20809724 10.3171/2010.3.SPINE09459

[30] Laufer I, Zuckerman SL, Bird JE, et al.: Predicting neurologic recovery after surgery in patients with deficits secondary to MESCC: Systematic review. Spine (Phila Pa 1976) 2016;41 Suppl 20:S224–S230 10.1097/BRS.0000000000001827 PMC5581189 27488300

